# Discovery of genes implicated in whirling disease infection and resistance in rainbow trout using genome-wide expression profiling

**DOI:** 10.1186/1471-2164-9-37

**Published:** 2008-01-24

**Authors:** Melinda R Baerwald, Amy B Welsh, Ronald P Hedrick, Bernie May

**Affiliations:** 1 Department of Animal Science, University of California Davis, Davis, CA, USA; 2 Department of Biological Sciences, SUNY Oswego, Oswego, NY USA; 3 Department of Medicine and Epidemiology, School of Veterinary Medicine, University of California Davis, Davis, CA, USA

## Abstract

**Background:**

Whirling disease, caused by the pathogen *Myxobolus cerebralis*, afflicts several salmonid species. Rainbow trout are particularly susceptible and may suffer high mortality rates. The disease is persistent and spreading in hatcheries and natural waters of several countries, including the U.S.A., and the economic losses attributed to whirling disease are substantial. In this study, genome-wide expression profiling using cDNA microarrays was conducted for resistant Hofer and susceptible Trout Lodge rainbow trout strains following pathogen exposure with the primary objective of identifying specific genes implicated in whirling disease resistance.

**Results:**

Several genes were significantly up-regulated in skin following pathogen exposure for both the resistant and susceptible rainbow trout strains. For both strains, response to infection appears to be linked with the interferon system. Expression profiles for three genes identified with microarrays were confirmed with qRT-PCR. *Ubiquitin-like protein 1 *was up-regulated over 100 fold and *interferon regulating factor 1 *was up-regulated over 15 fold following pathogen exposure for both strains. Expression of *metallothionein B*, which has known roles in inflammation and immune response, was up-regulated over 5 fold in the resistant Hofer strain but was unchanged in the susceptible Trout Lodge strain following pathogen exposure.

**Conclusion:**

The present study has provided an initial view into the genetic basis underlying immune response and resistance of rainbow trout to the whirling disease parasite. The identified genes have allowed us to gain insight into the molecular mechanisms implicated in salmonid immune response and resistance to whirling disease infection.

## Background

Whirling disease was first described among farmed rainbow trout (*Oncorhynchus mykiss*), a native North American salmonid species, introduced to Germany as a food fish in the late 1800s [1]. Whirling disease is associated with systemic infections by the myxozoan *Myxobolus cerebralis*, a parasite with presumed origins among salmonid fish in Eurasia [2,3]. Rainbow trout are highly susceptible to whirling disease and the introduction of the parasite to the U.S.A. in the 1950s had immediate economic impacts on salmonid hatcheries in both eastern and western states [2]. The parasite has a broad worldwide distribution and has been identified in 25 states in the U.S.A. where salmonid fish are present [4]. Salmonid hatcheries throughout the U.S.A have suffered drastic economic losses due to whirling disease outbreaks. The disease has more recently been recognized as the principal cause of major population declines among wild rainbow trout populations in the intermountain region of the U.S.A with serious negative impacts on sportfishing and allied industries [5-7]. Additionally, concerns continue over the potential negative ecologic impacts of whirling disease on wild salmonid populations, particularly threatened or endangered salmonids such as bull trout (*Salvelinus confluentus*), cutthroat trout (*Oncorhynchus clarki*), and steelhead (*Oncorhynchus mykiss*) [8,9].

*Myxobolus cerebralis *has a complex life cycle that includes two alternate hosts, a salmonid fish and an oligochaete worm, *Tubifex tubifex *[10,11]. Infection in the salmonid host begins when microscopic waterborne actinospore stages of *M. cerebralis *are released from the worm and contact the skin of the fish host. Actinospores, also referred to as triactinomyxons for *M. cerebralis*, attach preferentially to fins and the buccal cavity where they release one or more of three coiled polar filaments which penetrate and then anchor them to the epidermis[12]. Within minutes the sporoplasm, which contains 64 internal cells, migrates from the triactinomxyon to deeper layers of the epidermis, an action that may be facilitated by parasite proteases [13-15]. Aggregates and single cells from the sporoplasm then begin mitotic replication within two h of initial infection, alternating between inter and intracellular locations, a process that may also depend upon parasite coded protease activity [15,16]. Over the next 10 h at water temperatures of 15°C, parasites within host epithelial cells further divide by the process of endogeny or cell within cell replication prior to release and then penetration of new host cells. Between 12 and 20 h post infection, the number of parasite cells present in the epidermis steadily declines until new stages are observed in the subcutis at 48 h. Degenerative stages observed in the epidermis between 12 and 20 h are suspected to be a result of the action of the host immune response, although the cellular and or humoral factors involved are not currently known. After a brief residence in the subcutis, parasite stages are presumed to migrate to proximal nervous tissues, initially in peripheral and then more central locations [16]. Migration and potential replication of parasite stages in nervous tissue ensues over the next 16 d with the first parasites exiting to invade cartilage observed at 20 d post infection [16]. Feeding on cartilage may induce a host inflammatory response that constricts the spinal cord, brain stem, and caudal nerves resulting in the erratic swimming behavior (whirling) and black tail observed among fish with acute whirling disease [17]. An additional impact of cartilage destruction are permanent deformities to the skeletal system that may increase vulnerability to predation and impair ability to forage for food [1]. The final developmental stages of the parasite in the fish are environmentally resistant spore stages (myxospores) which remained trapped in cartilage or bone [18]. Death of infected fish or ingestion by fish or avian predators releases myxospores from the fish tissues and they may be ingested by the second host, the benthic dwelling oligochaete *T. tubifex *[19]. A second developmental cycle then occurs under the mucosal lining of the intestine that results in the release of thousands of the actinospore (triactinomyxon) stages potentially over the entire lifetime of the individual oligochaete [20].

Susceptibility to whirling disease in U.S. rainbow trout strains is pervasive with only two of the tested native strains displaying any degree of resistance, which may be inconsistent and relatively moderate [21,22]. Hatchery rainbow trout in Germany (Hofer strain), however, have acquired a degree of resistance to whirling disease that is consistently much higher than any domestic rainbow strains and comparable to that of brown trout (*Salmon trutta*), which are native to Europe and typically asymptomatic following infection [23]. Laboratory tests comparing rainbow trout strains under the same environmental conditions and pathogen exposure indicate that the Hofer strain's ability to combat *M. cerebralis *infection has a genetic basis. Recently, controlled crosses of the Hofer strain and a susceptible strain (Colorado River rainbow trout [CRR]) demonstrated that resistance to whirling disease was inherited by progeny [24] and heritability estimates are currently underway.

The discovery of the resistant Hofer strain allowed us to conduct an intraspecific comparison of susceptible and resistant rainbow trout in order to gain insight into the genetic basis underlying whirling disease susceptibility for this species. Gene expression profiling, through the use of microarrays, is an extremely high-throughput method to discover specific genes and pathways involved in a disease phenotype without the bias of a candidate gene approach.

In this study, microarray analysis was used to examine expression changes in a resistant and susceptible strain of rainbow trout following exposure to *M. cerebralis*, the pathogen causing whirling disease. We have found several genes significantly up-regulated in both the resistant and susceptible strain that appear to be involved in host response to infection. We have also found a gene which is significantly up-regulated in the resistant but remains unchanged in the susceptible rainbow trout strain following pathogen exposure that is a likely candidate gene for involvement in conferring whirling disease resistance.

## Results and Discussion

Quantitative PCR conducted on caudal fin tissues at two hours post exposure to *M. cerebralis *demonstrated each fish strain had similar initial pathogen loads, although there was substantial variation between individual fish within each strain. The mean parasite copy numbers per host cell were 1.20 × 10^6 ^(SD 1.53 × 10^6^) for the Hofer and 1.04 × 10^6 ^(SD 0.91 × 10^6^) for the Trout Lodge. These mean values and standard deviations are similar to those obtained in additional studies of susceptible rainbow trout when examined at early time points post TAM exposure (unpublished data).

In order to study genes involved in whirling disease response, resistant and susceptible rainbow trout strains were exposed to *M. cerebralis *and RNA from skin tissue was converted to cDNA and hybridized onto microarrays. Relative gene expression for exposed and unexposed controls for each strain was compared and the list of differentially expressed genes for both strains is found in Table 1. A combined total of 17 genes or features (14 annotated genes, 3 unknown features) were differentially expressed in one or both strains following pathogen exposure and are involved with rainbow trout infection response to whirling disease exposure. Several of these genes were found in different locations on the array as unique expressed sequence tag (EST) clones and their repeated presence on the significance gene lists provides additional support for their involvement in the whirling disease phenotype. The small number of genes found potentially indicates that only a few genes contribute to the phenotypic differences found between resistant Hofer and susceptible Trout Lodge, at least in terms of differential gene expression, during early disease progression in the skin. In the microarray statistical analysis, when the delta value was adjusted even slightly lower, the FDR estimate increases from 0% to ~78%. Since increasing the FDR cut-off to such a high percentage would dramatically reduce power, we chose to leave the gene list small with an estimated FDR of 0%. This type of dramatic increase in FDR estimation is additional support that there are not many genes differentially expressed in response to whirling disease infection for our chosen tissue and time points.

**Table 1:** EST clones significantly up-regulated in resistant and susceptible rainbow trout strains 24 hours after *Myxobolus cerebralis *exposure using a Wilcoxon test.

**Strain**	**Gene**	**Accession**	**Fold Change**
H	Ubiquitin-like protein 1*	CB499972	8.98
H	Ubiquitin-like protein 1*	CA064176	3.80
H	Interferon regulating factor 1*	CA063565	7.24
H	Interferon regulating factor 1*	CA058315	3.63
H	PPAR-α-interacting complex protein 285*	CA056844	7.88
H	Similar to interferon-inducible protein Gig2*	CA054168	5.82
H	Metallothionein B*	CB507722	4.36
H	Metallothionein B *	CB508872	3.08
H	Metallothionein B*	CA046225	2.72
H	Metallothionein B	CK990592	2.00
H	Metallothionein B*	CB510653	3.55
H	Interferon regulatory factor 7*	CB500977	4.36
H	Cyclin-dependent kinase 4 inhibitor B	CA044251	3.12
H	Cyclin-dependent kinase 4 inhibitor B	CB499959	2.66
H	Beta-2 microglobulin precursor	CN442516	2.51
H	Beta-2 microglobulin precursor	CB498391	2.23
H	Beta-2 microglobulin precursor	CB496576	2.12
H	Beta-2 microglobulin precursor	CB505897	2.04
H	Beta-2 microglobulin precursor	CA043324	2.02
H	Proteasome subunit beta type 8 precursor	CB496486	2.39
H	Interferon-induced 35 kDa protein homolog	CB493302	2.38
H	Haptoglobin precursor	CA038906	2.20
H	VHSV-induced protein	CB498971	2.07
H	Neighbor of COX-4	CB517140	2.01
H	Unknown	CA050082	2.71
H	Unknown	CA062379	2.70
H	Unknown	CB515535	2.41
TL	Ubiquitin-like protein 1*	CB499972	7.56
TL	Ubiquitin-like protein 1	CA064176	3.47
TL	Interferon regulating factor 1*	CA063565	4.81
TL	Interferon regulating factor 1	CA058315	2.80
TL	Interferon regulating factor 1	CA063863	2.55
TL	PPAR-alpha-interacting complex protein 285*	CA056844	3.31
TL	Similar to interferon-inducible protein Gig2	CA054168	3.20
TL	Similar to CC chemokine SCYA113	CB503743	2.26
TL	Unknown	CA062379	2.30

H = Hofer (resistant strain); TL = Trout Lodge (susceptible strain); * = In addition to being significant using the Wilcoxon rank sum, these genes are also significant using the modified *t*-test employed in SAM. *Q*-values < 1% were considered significant for both tests.

Different salmonid microarray platforms, such as those available from Oregon State University and Michigan State University, or different tissues and time points may produce additional candidate genes. A recent time course study used a candidate gene approach to identify four genes (TGF-β, IL-1β1, IL-1β2, and COX-2) that were significantly up-regulated by both Hofer and Trout Lodge in response to whirling disease infection [25]. These genes and their downstream effectors were not identified in the current microarray study, likely due to many differences in experimental design between the two studies (*e.g.*, pathogen exposure levels, tissue types, water temperatures, age of fish at exposure, etc.). While downstream effectors of these genes were present on the microarray, only one of the four genes (COX-2) was actually present on the microarray. It is our hope that future genome sequencing will enable the construction of more comprehensive microarray platforms for economically important aquaculture species, such as Atlantic salmon and rainbow trout.

All significant genes identified by the current microarray study were up-regulated following pathogen exposure for one or both strains. Therefore, it appears that both strains are undergoing transcriptional activation to defend against whirling disease infection and thus, are exclusively employing positive regulation for the genes examined in skin during early disease progression.

**Figure 1:**
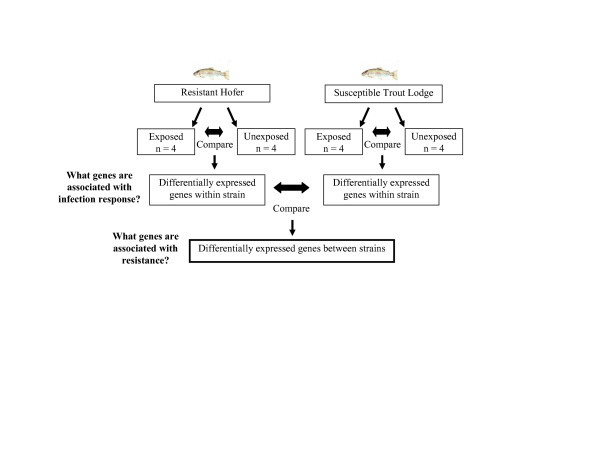
**Experimental approach for gene expression analysis**. Within strain comparisons were first conducted to identify genes responding to pathogen exposure. The expression profiles of these genes were then compared between resistant and susceptible strains to determine which genes are implicated in the whirling disease phenotype.

The normal caveats that apply for microarray studies (gene discovery is limited by transcripts on arrays, differences at transcriptional level may not cause phenotypic differences, results are dependent upon tissue type and time point chosen, etc.) apply for this study. Additionally, the comparison of two rainbow trout strains (i.e., resistant versus susceptible) added another layer of complexity to the analysis. We chose to not directly compare the two strains because there could be expression differences between them, due to divergence following strain isolation, that are unrelated to the whirling disease phenotype. With that in mind, the two strains were first compared entirely separately from each other to discover expression differences in response to pathogen exposure for each strain. Only the genes responding to infection, and therefore implicated in the whirling disease phenotype, were compared between the two strains for differential gene expression (Figure 1). A limitation of this approach to our study is that constitutively expressed transcripts which are differentially expressed between the two strains that contribute to the whirling disease phenotype cannot be identified.

### Microarray analysis of genes differentially expressed in the resistant Hofer strain in response to pathogen exposure

A total of 16 genes or features (13 annotated genes, 3 unknown features) were up-regulated in the resistant Hofer strain following pathogen exposure. All 13 annotated genes have been previously implicated in host immune response for other infectious diseases. *Viral Hemorrhagic Septicemia Virus *(*VHSV*) *induced protein *and *neighbor of COX-4 *are the only annotated genes without known molecular functions.

A common link between the majority of annotated genes with known molecular functions is an involvement in the interferon system. The interferon system is one of the first lines of host defense against invading pathogens for vertebrates (for review see [26]), including teleost fish (for review see [27]). Other economically important salmonid pathogens, such as infectious pancreatic necrosis virus and infectious salmon anaemia virus have been found to activate both type I and type II interferon (IFN) responses in the Atlantic salmon host following infection [28]. Interferons are cytokine proteins that are secreted following infection and play a critical role in both innate and adaptive immunity. The IFN system has been most widely researched in mammals and studies have found that type I IFN (mammalian IFN-α/β) are secreted by the pathogen-infected cells as part of a rapid initial immune response while Type II IFN (mammalian IFN-γ) is secreted by natural killer (NK) and T cells and plays a more central role in the second wave of immune response. To cope with the myriad of host infections, the interferon system is highly complex and involves the regulation of hundreds of genes [29,30]. Specifically, type I IFN acts to increase MHC class I expression for antigen presentation [31], promote T cell survival [32], inhibit cell proliferation [33], mediate apoptosis [26], and increase NK cell activity [34]. Type II IFN acts to increase both MHC class I and II expression for antigen presentation [29], stimulate macrophages to kill engulfed pathogens [35], induce apoptosis [36], and regulate leukocyte-endothelium interactions [37] in addition to many other immune-related activities.

It is informative to examine the functional roles of each gene's encoded protein specifically to better understand the part each plays, both individually and as interconnected components, in host immune response. Expression of the interferon-induced 35 kDa protein is induced by IFN and it is involved in cytokine signalling [38]. Interferon regulatory factor 1 (IRF-1) and interferon regulatory factor 7 (IRF-7) are transcription factors that induce expression of IFN responsive genes [39,40]. Additionally, IRF-1 is involved in apoptosis and cell cycle regulation related to tumor suppression [41]. Similarly, cyclin-dependent kinase 4 inhibitor B (p15-INK4b) plays a role in apoptosis[42], cell cycle regulation [42], and tumor suppression [43] and can be induced by the cytokine TGF-β [44]. Gig2 is an interferon-inducible protein that is likely part of the JAK-STAT signal transduction pathway [45]. Ubiquitin and the proteasome subunit beta type 8 precursor are both members of the ubiquitin-proteasome system (for review see [26]), which serves to degrade proteins via proteolysis. These degraded proteins can originate from an invading pathogen and are displayed on MHC class I proteins. The beta-2-microglobulin is an integral component of MHC class I proteins and is therefore involved in antigen processing and presentation to cytotoxic T cells [46]. Haptoglobin binds hemoglobin and limits its availability to infectious bacteria, thus preventing bacterial proliferation in a wound [47]. The PPAR-α-interacting complex protein 285 is a transcriptional co-activator with helicase activity [48] and has sequence similarity to a rainbow trout VHSV-induced protein. Gene expression of metallothionein B (MT-B) is induced by several metal ions [49], cytokines [50-52], and stress hormones [53-55]. MT proteins are believed to play diverse functional roles in inflammation, immune response, apoptosis, tumor suppression, and detoxification (for reviews see [55,56]).

### Microarray analysis of genes differentially expressed in the susceptible Trout Lodge strain in response to pathogen exposure

A total of six genes or features (five annotated genes, one unknown feature) were up-regulated in the susceptible Trout Lodge strain following pathogen exposure. Only one of the significant genes for Trout Lodge, which has sequence similarity to *CC chemokine SCYA113*, was not also differentially expressed in Hofer in response to pathogen exposure. The *CC chemokine SCYA113 *gene is a member of the CC chemokine family, which guides leukocytes to sites of infection and inflammation (for review see [57]). The fewer number of significant genes found for Trout Lodge relative to Hofer may indicate a decrease in transcriptional activation for this susceptible strain. There is, however, likely some degree of overlap in both strains' response to pathogen exposure due to the fact that several genes were up-regulated in both Hofer and Trout Lodge (i.e., *ubiquitin-like protein 1*, *IRF-1*, and *PPAR-α-interacting protein Gig2*). A critical phase in the early stages of *M. cerebralis *infection in trout is invasion and intracellular replication, processes that begin as early as one hour post exposure to triactinomyxons [16]. A role for accumulated ubiquinated proteins in the lysosome in the killing of *Mycobacterium tuberculosis *has recently been described that has implications for a range of intracellular infections [58] and some similar responses to infection may be occurring for both resistant and susceptible strains.

### Microarray analysis of genes differentially expressed between resistant and susceptible strains in response to pathogen exposure

Of the genes differentially expressed in response to pathogen exposure for both strains, only metallothionein B shows a statistically significant difference in expression between the two strains (Table 2). *MT-B *was found to be up-regulated in the resistant Hofer strain following pathogen exposure but remained unchanged in the susceptible Trout Lodge strain.

**Table 2:** EST clones differentially expressed between resistant and susceptible rainbow trout strains in response to *Myxobolus cerebralis *exposure.

**Gene**	**Accession**	**Fold Change**	**p-value**
Metallothionein B	CB507722	5.20	0.002
Metallothionein B	CA046225	4.06	0.004
Metallothionein B	CB508872	3.52	0.007
Metallothionein B	CB510653	3.84	0.004

Fold changes are computed from log ratio values (Hofer/Trout Lodge) and represent the relative increase in expression levels for Hofer versus Trout Lodge following pathogen exposure.

As previously noted, metallothionein has been implicated in a broad range of functional capacities, including inflammatory and immune responses. Several cytokines can induce metallothionein expression including IFN [59-61], interleukin-1 [50], interleukin-6 [51], and tumor necrosis factor-α [52]. Metallothionein has been shown to mediate leukocyte chemotaxis and has been hypothesized to serve as an early "danger signal" during times of stress or infection to activate an immune response [62]. The functional similarities between metallothionein and CC chemokine SCYA113, at least in terms of leukocyte chemotaxis, are certainly of interest since these genes displayed quite distinct expression profiles. *Metallothionein *was up-regulated in the resistant Hofer strain and *CC chemokine SCYA113 *was up-regulated in the susceptible Trout Lodge strain (although *CC chemokine SCYA113 *did not pass the significance cut-off to be considered differentially expressed between the two strains). This distinction between two genes, capable of similar biological roles, may indicate that leukocyte movements to, and their activities once at, the infection site are key factors in determining resistance versus susceptibility to whirling disease. Evaluations by light microscopy and qPCR for *M. cerebralis *genomic DNA of Hofer and Trout Lodge rainbow trout exposed to triactinomyxons demonstrates Hofer more efficiently eliminates invading parasites in the skin (M. Adkison, pers. comm.). While the parasite effectively penetrates the epidermis in both strains, significantly fewer parasites survive the migration from the skin to the nerves as evaluated at 10 d post exposure. A role for host immune factors in the elimination of invading parasites, even in susceptible rainbow trout strains, is suggested by several prior light and electron microscopy studies that demonstrate an increase in degenerative stages in the skin beginning as early as 12 h and then their elimination by 24 h post-exposure to triactinomyxons [12,16,63].

The difference in metallothionein expression may be due to an alternative immune response pathway since the protein has known involvement in diverse functional capacities. For instance, metallothionein's role as a zinc-finger transcriptional regulator [64] may dramatically alter the expression profiles between resistant and susceptible rainbow trout. All biological roles of this diverse protein should be considered when examining the complexities of host immune response. Additionally, upstream regulators of metallothionein expression could be the true underlying cause of the whirling disease phenotype since a gene expression study alone cannot determine if a gene is directly contributing to a phenotype (i.e., cause versus downstream effect).

### Validation of microarray results by qRT-PCR

**Figure 2:**
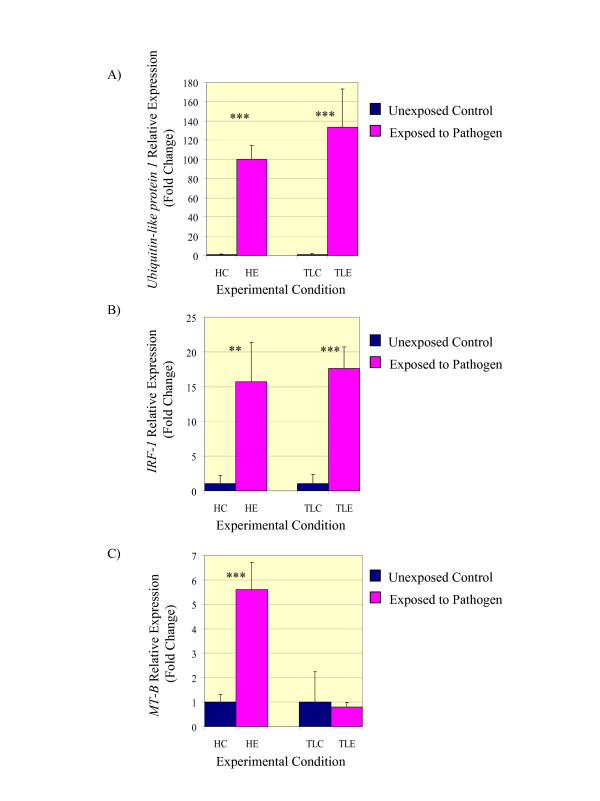
**Quantitative RT-PCR expression results for genes responding to *Myxobolus cerebralis *infection in the skin tissue of both strains**. A) *Ubiquitin-like protein 1 *is 100 – 134 fold up-regulated following pathogen exposure for both strains. B) *Interferon regulating factor 1 *(*IRF-1*) is up-regulated 16 – 18 fold up-regulated for both strains. C) *Metallothionein B *(*MT-B*) is up-regulated over 5 fold in the resistant Hofer but is unchanged in the susceptible Trout Lodge strain. Asterisks represent significance (** = *P *< 0.005; *** = *P *< 0.001). Abbreviations: HC = Hofer Control; HE = Hofer Exposed; TLC = Trout Lodge Control; TLE = Trout Lodge Exposed.

Quantitative RT-PCR (qRT-PCR) confirmed the microarrays results for two of the genes up-regulated in both Hofer and Trout Lodge following infection, *ubiquitin *and *IRF-1*, and the *metallothionein *gene (*MT-B*), which was up-regulated in Hofer but remained unchanged in Trout Lodge following infection (Figure 2). The qRT-PCR results for *IRF-1 *and *metallothionein *were quite similar to the microarray results for each gene, in terms of relative expression changes in response to infection. *MT-B *was found to once again be significantly up-regulated in the resistant Hofer strain following pathogen exposure but remained unchanged in the susceptible Trout Lodge. This difference in *MT-B *gene expression between the two strains was statistically significant (*P *~ 0.001). The relative degree of up-regulation for ubiquitin following pathogen exposure was considerably higher in the qRT-PCR (~9 – 17 fold greater up-regulation in qRT-PCR versus microarrays). Many other studies have also observed this pattern of greater sensitivity in qRT-PCR versus microarray results (for examples see [65,66], which is often attributed to the more gene-specific optimized conditions of the qRT-PCR approach.

Given the high degree of statistical support and biological relevance of the candidate genes, we believe this study provides initial insight into rainbow trout genes and pathways responding to whirling disease infection and identifies the first candidate genes for whirling disease resistance.

### Potential future studies

While the interferon system appears to be a likely candidate system for further study, many of the significant genes are found in alternative pathways and have distinct roles and functions in other systems. Furthermore, it is increasingly apparent that epistatic interactions and the interplay between pathways/networks previously classified as discrete can have enormous phenotypic effects on quantitative traits [67]. Multiple avenues of research should be examined in future studies, using the candidate genes presented here as an initial guide, due to the complex relationships between hosts and pathogens. For instance, the migration of leukocytes and their subsequent activity in the skin are likely a critical part of the early immune and inflammatory host response after pathogen infection. Additionally, it is quite feasible that the difference in metallothionein expression is due to an alternative immune response pathway since the protein has known involvement in diverse functional capacities. For instance, metallothionein's role as a zinc-finger transcriptional regulator [64] may dramatically alter the expression profiles between resistant and susceptible rainbow trout. All biological roles of this diverse protein should be considered when examining the complexities of host immune response. Finally, upstream regulators of metallothionein expression could be the true underlying cause of the whirling disease phenotype since a gene expression study alone cannot determine if a gene is directly contributing to a phenotype (i.e., cause versus downstream effect). The expression profiles of a variety of metallothionein upstream regulators, such as cytokines and *metal transcription factor *(*MTF-1*), could be evaluated in a time course study during early disease progression to identify additional candidate genes. A QTL mapping approach could also be used to identify particular chromosomal regions directly contributing to the disease phenotype.

## Conclusion

The present study has provided the first examination into the genetic basis underlying rainbow trout's immune response and resistance to the whirling disease pathogen. Several genes were significantly up-regulated in skin following pathogen exposure for both the resistant Hofer and susceptible Trout Lodge rainbow trout strains. For both strains, response to infection appears to be linked with the interferon system. *Metallothionein B *is differentially expressed between the resistant and susceptible strains and is a good candidate for future whirling disease resistance studies. The identified genes have allowed us to gain initial insight into the molecular mechanisms involved in a salmonid host's immune response and resistance to whirling disease infection.

## Methods

### Animal care, pathogen exposure, and RNA preparation

Hofer and Trout Lodge rainbow trout strains were reared in 35 gallon aquaria with 15°C flow-through well water for nine weeks post-hatch, with each fish weighing approximately 6.5 grams prior to pathogen exposure. Individuals from each strain (n = 60) were exposed to 2,000 triactinomyxons (TAMs) per fish for one hour. Additional fish (n = 60) from both strains served as unexposed controls, which were treated identically to exposed fish at all experimental stages other than their lack of pathogen exposure. Fish were then kept under standard aquaculture conditions until euthanized. TaqMan PCR for the quantitative evaluation of genomic parasite DNA was employed to confirm that fish in both the Hofer and Trout Lodge groups received equal amounts of parasite exposure. At two hours post TAM exposure, 6 fish in each exposed group were removed and euthanized with an overdose of benzocaine at a concentration of 500 mg/L. Caudal fins were removed posterior to the peduncle and used as the tissue for a quantiative TaqMan assay following procedures described by Kelley et al. [68].

Microarray studies examining skin four hours after pathogen exposure did not identify any genes differentially expressed between Trout Lodge and Hofer strains (data not shown). Therefore, we chose a later time point (24 hours after exposure) so that early host immune response was more likely to be fully underway and significant expression changes could be detected. After the 24 hour incubation period, all fish were euthanized with an overdose of benzocaine at a concentration of 500 mg/L. Each fish was euthanized individually and the caudal fin (largely comprised of skin tissue) was removed posterior to the peduncle. The fin was immediately placed into 2× Nucleic Acid Purification Lysis Solution supplied with ABI's TransPrep Chemistry kit (Applied Biosystems, Foster City, CA) to stop further gene expression changes. Total RNA was extracted from the fin of each individual using the ABI Prism™ TransPrep system with the ABI Prism™ 6100 Nucleic Acid PrepStation according to manufacturer instructions. RNA quality was assessed by agarose gel electrophoresis and RNA concentrations were measured using a ND-1000 spectrophotometer (NanoDrop Technologies, Wilmington, DE).

Starting total RNA yields were not sufficient for microarray hybridizations due to the small amount of caudal fin tissue present on these young fish. Therefore, 250 – 1000 ng of total RNA was used as the starting material to create amplified RNA (aRNA) indirectly labeled with Cy3 or Cy5 fluorescent dyes (GE Healthcare, Buckinghamshire, UK) using the Amino Allyl MessageAmp™ II aRNA Amplification kit according to manufacturer instructions (Ambion, Austin, TX).

### Microarray hybridization and data analysis

Salmonid cDNA microarrays (GRASP16k v2.0) were obtained from consortium for Genomic Research on Atlantic Salmon (cGRASP) and details of microarray development and fabrication can be found in von Schalburg et al. [69]. These arrays contain 13,421 Atlantic salmon and 2,576 rainbow trout cDNA features and have been successfully used for several previous rainbow trout gene expression studies [70-73]. For each rainbow trout strain, competitive hybridization was conducted on every array using equal amounts (8 μg) of differentially labeled aRNA from one control fish and one exposed fish. Four biological replicates were performed for each experimental condition and dye-sample coupling was swapped between biological replicates in a balanced block design.

Prehybridization washes for all microarrays included: 2 × 5 min in 0.1% SDS, 5 × 1 min in NANOpure H_2_O with 0.5 mM dithiothreotol, 1 min in near boiling nanopure H_2_O, centrifugation for 2 min at 1500 RPM. To reduce background, the microarrays were next incubated for 90 min in 5 × SSC, 0.1% SDS, 3% BSA (Fraction V) at 49°C, washed 3 × 20 s in nanopure H_2_O, and dried by centrifugation for 5 min at 1500 RPM. The labeled aRNA samples were competitively hybridized to microarrays prewarmed to 49°C for 16 hours in a formamide-based buffer (Genisphere, Hatfield, PA) with LNA dT blocker (Genisphere). Posthybridization washes for all microarrays included: 1 × 10 min in 2 × SSC, 0.1% SDS prewarmed to 49°C, 2 × 5 min in 2 × SSC, 0.1% SDS at room temperature, 2 × 5 min 1 × SSC at room temperature, 2 × 5 min 0.1 × SSC at room temperature. Slides were then dried by centrifugation and immediately scanned using an Agilent G2565BA Microarray Scanner (Agilent Technologies, Santa Clara, CA).

Data underwent local background subtraction and LOWESS normalization using Agilent's Feature Extraction software. Raw and processed gene expression data have been deposited into the NCBI Gene Expression Omnibus [74] (series GSE8631) and are in compliance with MIAME guidelines. The Significance Analysis of Microarrays (SAM) software package [75] was used to identify differentially expressed genes between exposed and unexposed control fish for each rainbow trout strain. Both a Wilcoxon rank sum and a modified *t*-test were conducted with 1,000 permutations and the minimum fold change cut-off was set to 2.0 up- or down-regulated. A false discovery rate (FDR) of 0.00% was estimated for both strains. To determine statistically significant differences between the Hofer and Trout Lodge strains, a Welch's *t*-test (P-value < 0.01) was implemented in Microsoft Excel between the log ratios (exposed/control) for each strain for all genes that were significant for at least one strain in the SAM program.

### Quantitative RT-PCR

Microarray expression results were validated by qRT-PCR for several identified genes. Prior to qRT-PCR, 80 ng of total RNA was reverse transcribed from each biological replicate used for the microarray study along with two additional samples (total n = 6 per experimental condition) using the QuantiScript Reverse Transcriptase kit (Qiagen, Valencia, CA) according to manufacturer instructions. In contrast to the microarray experiments, the template RNA was not amplified before cDNA synthesis. EST clone sequences from the cGRASP microarray were used to design primers for genes undergoing validation, along with a β-actin reference gene used for normalization, with Primer3 software [76] and the sequence for each primer pair is shown in Table 3. The Quantitect™ SYBR^® ^Green RT-PCR kit (Qiagen) was used according to the manufacturer's instructions except the final PCR volume was reduced to 25 μl. The PCR conditions used on a Chromo4 Real Time PCR Detection System (Bio-Rad, Hercules, CA) were as follows: HotStarTaq DNA polymerase activation at 95°C for 15 min, 45 cycles of 15 s denaturation at 94°C, 30 s annealing at 58°C, 30 s extension at 72°C, followed by a melting curve to ensure that a single PCR product was produced for each reaction. For each gene, the relative amount of gene expression was calculated using the ΔΔC_T _method [77] and significance was determined using a nonparametric Mann-Whitney U test and multiple linear regression in JMP.

**Table 3:** Primer sequences for qRT-PCR validation.

**Gene**	**Accession**	**Forward primer**	**Reverse primer**
Interferon regulating factor 1	CA063565	TCGTCCGGGAATATACTGCT	CGTTGCCAGAACATCACATC
Ubiquitin-like protein 1	CB499972	AAAGCCAATATCAGCCATGC	ACCTGAAGCCCTTAACTTA
Metallothionein-B	CB507722	ACACACACAGCCTGAAGCAC	TGAATAAAGAAGCGCGATCA
β-actin	AF157414	GCCCTCTTCCAGCCCTCCTTCCT	TGCCGGGGTACATGGTGGTTCCT

## Authors' contributions

MRB participated in study conception and design, conducted gene expression experiments and data analysis, and drafted the manuscript. ABW participated in study design, data analysis, and manuscript revision. RPH and BPM participated in study conception and design, supervision of research activities, and manuscript revision. All authors read and approved the final manuscript.
